# Probiotics *Bifidobacterium lactis* M8 and *Lactobacillus rhamnosus* M9 prevent high blood pressure via modulating the gut microbiota composition and host metabolic products

**DOI:** 10.1128/msystems.00331-23

**Published:** 2023-10-19

**Authors:** Yong Zhang, Tingting Zheng, Da Ma, Peng Shi, Heping Zhang, Jun Li, Zhihong Sun

**Affiliations:** 1 Key Laboratory of Dairy Biotechnology and Engineering, Ministry of Education, Inner Mongolia Agricultural University, Huhhot, China; 2 Key Laboratory of Dairy Products Processing, Ministry of Agriculture, Inner Mongolia Agricultural University, Huhhot, China; 3 School of Chemistry and Biological Engineering, University of Science and Technology Beijing (USTB), Beijing, China; 4 Shenzhen Research Institute, City University of Hong Kong, Shenzhen, China; 5 Department of Infectious Diseases and Public Health, Jockey Club College of Veterinary Medicine and Life Sciences, City University of Hong Kong, Hong Kong, China; 6 School of Data Science, City University of Hong Kong, Hong Kong, China; Universita degli Studi di Napoli Federico II, Portici, Italy

**Keywords:** probiotics, efficacy, hypertension, metagenome, metabolome

## Abstract

High-fructose intake is one of the high-risk factors for hypertension. Several probiotics have been reported to reduce high blood pressure (BP) via modulating gut microbiota, but the mechanism of the antihypertensive effect of probiotics is insufficiently understood. Herein, we investigated the antihypertensive effect of *Bifidobacterium lactis* M8 and *Lactobacillus rhamnosus* M9 based on the integrative analysis of gut metagenome and serum metabolome in high-fructose-treated mice. After 16-week intervention, M8 and M9 significantly reduced the median blood pressure by 16.92% and 15.39% in SBP, and 18.56% and 20.62% in DBP, respectively. Metagenomic analysis revealed that the increased *Lawsonia* and *Pyrolobus*, and reduced *Alistipes* and *Alloprevotella* levels were tightly correlated with lowered BP. Functionally, the decreased pathways of “base excision repair” (BER) and “D-glutamine and D-glutamate metabolism” were associated with BP reduction. Metabolomics data analysis further revealed that the probiotic interventions regulated vascular smooth muscle contraction, serotonergic synapse, cholinergic synapse, and lipid and vitamin metabolism in BP control. Additionally, association analyses indicated a strong negative correlation between *Alistipes* and “steroid hormone biosynthesis,” suggesting that *Alistipes* could affect blood pressure by changing steroid hormone levels. In conclusion, our findings indicate that the probiotic efficacies in alleviating hypertension are linked to specific gut microbes and metabolic pathways, which provide a potential mechanistic understanding of probiotics modulated blood pressure, paving the way for future assessment of the therapeutic potential of probiotics in hypertension management.

**IMPORTANCE:**

Elevated blood pressure affects 40% of the adult population, which accounts for high cardiovascular disease risk and further high mortality yearly. The global understanding of the gut microbiome for hypertension may provide important insights into the prevention. *Bifidobacterium lactis* M8 and *Lactobacillus rhamnosus* M9 originated from human breast milk, were able to decrease blood pressure, and modified metabolites in a high fructose-induced elevated blood pressure mouse model. Moreover, we found there was a close relationship between unexplored gut microbes and elevated blood pressure. Also, subsequently, the cross-link was explored among gut microbes, metabolites, and some metabolic pathways in gut microbial environment through introducing novel prediction methodology and bioinformatic analysis. It allowed us to hypothesize that probiotics can prevent elevated blood pressure via gut microbiota and related metabolism.Thus, utilization of dietary strategies (such as probiotics) to maintain the blood pressure level is of crucial importance.

## INTRODUCTION

Hypertension is one of the most common chronic diseases affecting the human life span (1). It was estimated that 31.1% of adults (1.39 billion) worldwide had hypertension in 2010 (2). Increased sugar consumption, especially dietary fructose syrup intake, is responsible for the increased incidence of hypertension (3). Many studies have indicated that high fructose may increase the risk of hypertension through a variety of mechanisms, including increased salt retention, insulin resistance, decreased renal nitric oxide production, and, recently, altered gut microbial composition (4–6). Some studies have reported that the consumption of high fructose may lead to decreased *Bacteroides* and elevated Firmicutes levels (7). *Bacteroides* spp. are known to produce several short-chain fatty acids (SCFAs), which are involved in blood pressure (BP) control via G protein-coupled sensory receptors (6, 8).

Massive evidence-based studies have revealed that probiotics have a positive impact on human health through mediating the number and diversity of gut microbiota and improving the immune system response (9). Currently, researchers are actively investigating the roles of probiotics in various diseases. Different probiotics have been found to deliver treatment effects under different clinical conditions. For example, *Lactobacillus acidophilus* and *Lactobacillus rhamnosus* exhibited efficacies in mitigating symptoms of acute gastroenteritis (10, 11). Another clinical study indicated that multispecies probiotics showed anti-inflammatory effects in irritable bowel syndrome therapy (12).

Notably, the protective effect of probiotics on hypertension has been studied. Hsu et al. found that *Lactobacillus casei* could protect against hypertension, which was related to reduced plasma acetate levels and decreased renal Olfr78 expression (13). Other studies indicated that probiotics could improve endothelial dysfunction via releasing converting enzyme inhibitory peptides and impairing lipopolysaccharide signaling, ultimately reducing BP (14, 15). Moreover, probiotics may alleviate high blood pressure by altering the gut microbiota, such as inhibiting pathogenic bacterial colonization through modulating the abundances of metabolites (16).

Recently, integrative analyses of fecal metagenomics and serum metabolomics have begun to bridge the gap between the microbiota and host metabolic activity in response to probiotic interventions, but the mechanism of the probiotics’ antihypertensive effect has been inadequately investigated via such analytical methods. Therefore, in this study, by using shotgun metagenomic sequencing and untargeted mass spectrometry (MS)-based serum metabolomics, we evaluated the effect of two probiotics, *Bifidobacterium lactis* M8 and *Lactobacillus rhamnosus* M9, on the alterations in gut microbiome and serum metabolome and correlated them with BP. Several microbial taxa and functional units were identified as signatures statistically associated with BP. Furthermore, we revealed the interactions between genus and metabolic function, elucidating the potential mechanism by which probiotics alleviate hypertension.

## RESULTS

### Probiotic treatments prevent the high fructose-induced high BP level

**Fig 1:**
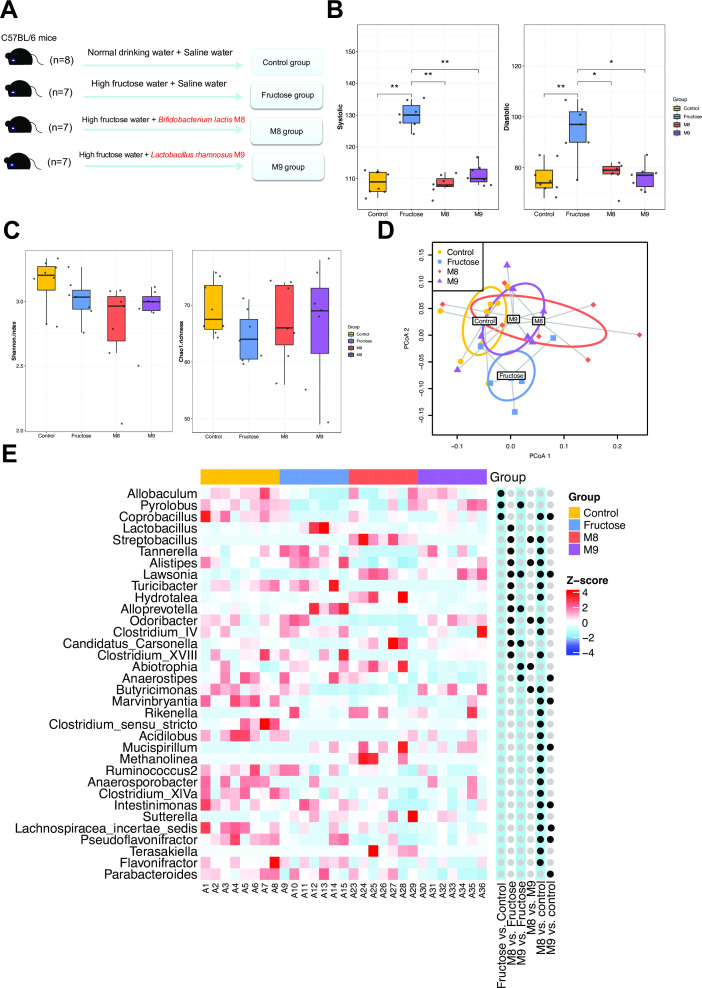
Probiotics reduce blood pressure level through modulating the composition of gut microbiota. (A) The experiment design of this study; (B) the blood pressure level (left: SBP, right: DBP) after 16-week intervention; Wilcoxon test was used to detect the significant change in blood pressure between groups: *P < 0.05, **P < 0.01. (C) Microbial diversity (left: Shannon index, right: Chao1 richness) of gut microbial community at genus level after 16-week intervention; (D) principal coordinates analysis (PCoA) analysis indicated the difference in overall composition of gut microbial community among the groups using weighted UniFrac distance; (E) heatmap of genus showing differential abundance in at least one pairwise comparison using Wilcoxon test. For visualization, the abundance levels were log-transformed then centered and scaled to a mean of 0 and standard deviation of 4. Black dots indicate the significance level of the comparison (false discovery rate (FDR) <0.25).

We designed a 16-week intervention study (Fig. 1A) using mice to investigate whether and why the probiotics *B. lactis* M8 and *L. rhamnosus* M9 are capable of reducing the fructose-induced high BP levels. Twenty-nine mice were divided into four groups fed (i) normal drinking water (the “control group”); (ii) high-fructose water (15% [wt/vol]) plus normal saline intragastric administration (the “fructose group”); either (iii) high fructose plus *B. lactis* M8 (4 × 10^9^ CFU/day) in intragastric saline (the “M8 group”) or (iv) high fructose in drinking water plus *L. rhamnosus* M9 (4 × 10^9^ CFU/day) in intragastric saline (the “M9 group”) (Fig. 1A). The BP levels were measured at multiple timepoints, including day 0 and weeks 4, 10, and 16 (Table S1; Fig. S1). The fructose intervention introduced significantly higher systolic blood pressure (SBP) and diastolic blood pressure (DBP) levels in the fructose group than the control group (SBP: Wilcoxon test, *P* = 0.0014; DBP: Wilcoxon test, *P* = 0.0053; Table S1; Fig. 1B). The SBP and DBP levels of the M8 and M9 groups were significantly lower than that of the mice in the fructose group (SBP: M8 vs fructose: Wilcoxon test, *P* = 0.00211; M9 vs fructose: Wilcoxon test, *P* = 0.00207; DBP: M8 vs fructose: Wilcoxon test, *P* = 0.021; M9 vs fructose: Wilcoxon test, *P* = 0.010). The median SBP decreased by 16.92% and 15.39%, and the median DBP decreased by 18.56% and 20.62% in the M8 and M9 groups, respectively (Table S1; Fig. 1B). The results showed that probiotic interventions efficiently prevented both the high SBP and DBP induced by fructose in the two probiotics groups (Fig. 1B). We also found that no significant difference in BP level between the M8/M9 and the control groups (Wilcoxon test, *P* > 0.05), indicating that the probiotic interventions would retain the BP at a normal level under the high-fructose intervention.

### The effect of probiotics on the gut microbiota of high fructose-treated mice

We conducted shotgun metagenomic sequencing to uncover the links between the gut microbiota modulated by the probiotics and BP reduction. The taxonomic profiling identified by Ribosomal Database Project (RDP) classifier revealed that the microbial communities were dominated by Bacteroidetes (51.25%), Firmicutes (33.70%), and Proteobacteria (11.29%) in all four groups (Fig. S2; Table S2). Compared to the control group, there was an increase in Bacteroidetes and a decrease in Firmicutes in the fructose group (Wilcoxon test, *P* < 0.05), while in M8 and M9 groups, the abundances of Bacteroidetes recovered to almost the control level (M8 vs control: Wilcoxon test, *P* = 0.955; M9 vs control: Wilcoxon test, *P* = 0.694) (Fig. S2; Table S2).

The comparison of microbial diversity at the genus level revealed that the M8 and M9 groups had a slightly decreased Shannon alpha diversity (7.32% and 2.70% on average, respectively) than that in the fructose group, but the significance was not high (M8 vs fructose: Wilcoxon test, *P* = 0.383, M9 vs fructose: Wilcoxon test, *P* = 0.535) (Fig. 1C). Compared to the fructose group, slight but not significant increases (3.99% and 3.10% on average) in bacterial richness (Chao1 richness) were observed in the M8 and M9 groups (M8 vs fructose: Wilcoxon test, *P* = 0.369; M9 vs fructose: Wilcoxon test, *P* = 0.481) (Fig. 1C).

We further compared the overall composition difference of the gut microbial community among the four groups using weighted UniFrac distance. The overall gut community composition showed significant differences across the four treatment groups (Adonis test, *P* = 0.001). Moreover, we observed that the microbial communities of the probiotic-treated groups (M8 and M9) had more closer community structures to the control group than to the fructose group (mean pairwise distances: between M8 and control: 0.192, between M9 and control: 0.148, between M8 and fructose: 0.205, between M9 and fructose: 0.162), which indicated that the taxonomic variation of gut communities from the fructose-treated mice may partially recovered to the normal status (Fig. 1D).

We subsequently performed differential abundance analysis to identify genera whose relative abundance was significantly altered under probiotic treatment (Table S3; Fig. 1E). Three genera (*Lawsonia*, *Alloprevotella*, and *Candidatus Carsonella*) were differentially abundant in both comparisons of M8 vs fructose, and M9 vs fructose (Wilcoxon test, false discovery rate (FDR)-corrected *P* < 0.25). Moreover, compared to the fructose group, nine genera were only differentially abundant in the M8 group, including higher abundance of *Hydrotalea* and lower abundance of *Alistipes*, *Clostridium* IV, and *Clostridium*_XVIII. Conversely, three genera that had differential abundance were only detected in M9 vs fructose (Wilcoxon test, FDR-corrected *P* < 0.25) (Fig. 1E). Specifically, the M9 group had elevated levels of *Pyrolobus* and reduced levels of *Abiotrophia* and *Anaerostipes*.

At the species level, metagenomic reads were identified and significantly altered species by probiotic were listed (Table S4; Fig. S3). In comparison to the fructose treatment, 16 species had differential abundance (Wilcoxon test, FDR-corrected *P* < 0.1, mean abundance >0.01%) in the M8 group. The six species, including *Bacteroides dorei*, *Bacteroides vulgatus*, and *Bifidobacterium animalis*, were more abundant in the M8 group. The other 10 species, including *Alistipes shahii*, *Alistipes dispar*, and *Alistipes megaguti*, were less abundant in the M8 group, consistent with the significantly reduced levels of *Alistipes* identified by RDP. Conversely, compared to the fructose group, the M9 group harbored eight species that were differentially abundant. Among these differential species, four species were also differentially abundant when we compared M8 with fructose. Specifically, more abundant *Paenibacillus larvae*, less abundant *Lactobacillus reuteri*, and *Lactobacillus johnsonii* were found in the M8 and M9 group as compared to the fructose group. *Bacteroides dorei* was significantly increased in the M8 group but decreased in the M9 group. These probiotics-modulated species should be investigated further for their influence on BP.

We also identified the significant changes in the microbial pathways induced by the probiotic treatment. In the comparison of M8 vs fructose, 101 differentially abundant pathways were identified (Wilcoxon test, FDR-corrected *P* < 0.05). Of these pathways, 21 pathways increased in the M8 group and were mainly involved in signal transduction, cellular community, and immune system (Table S5). Other pathways depleted in the M8 group were associated with amino acid, carbohydrate, nucleotide, and lipid metabolism (Table S5). These results demonstrated that the M8 could influence functional capabilities of microbial communities extensively, accompanied with immune modulation. In contrast, no significant difference in microbial pathways was observed between M9 and fructose group.

### Identify microbial taxa associated with BP based on compositional balance analysis

**Fig 2:**
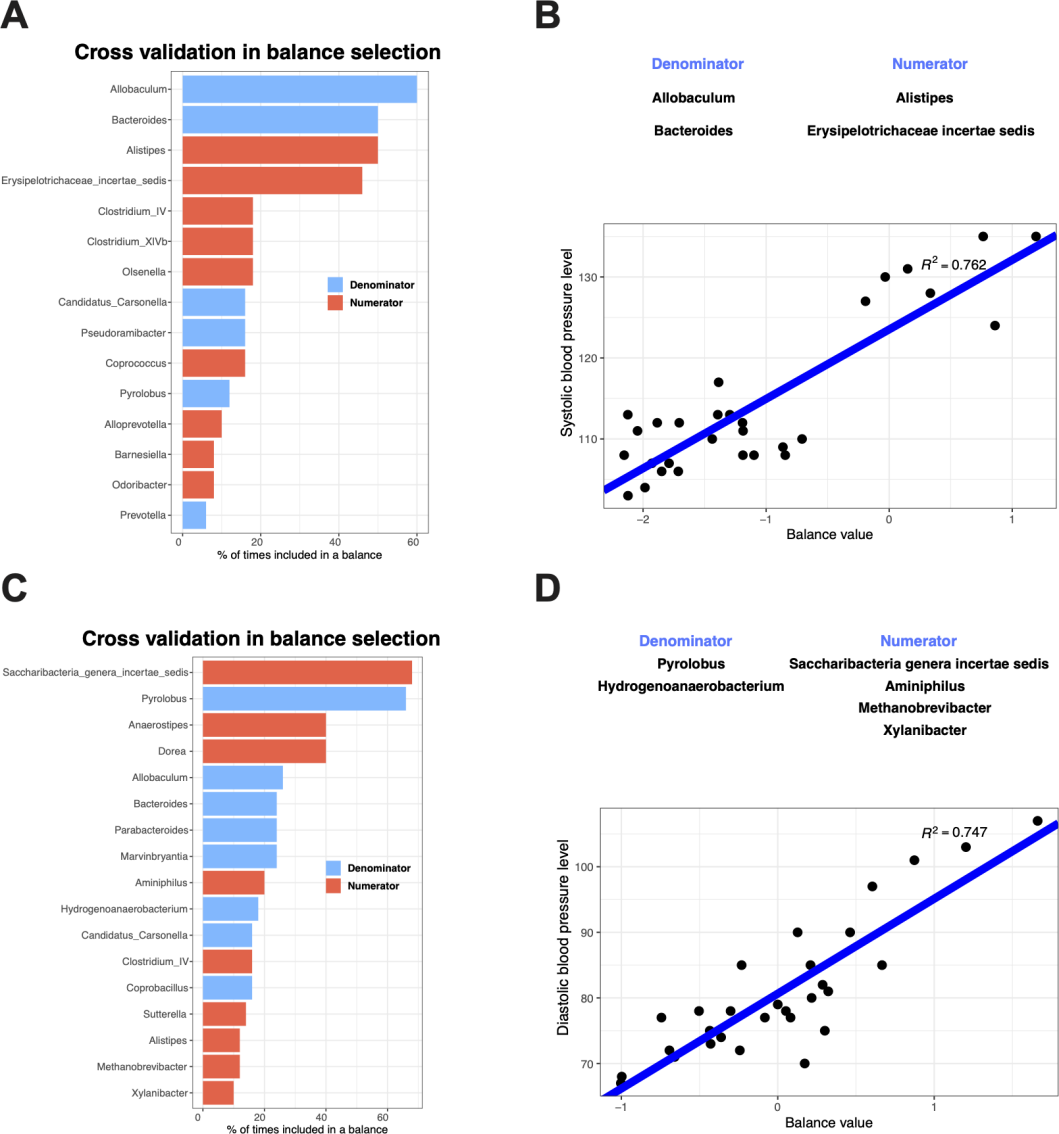
The microbial balance associated with SBP (A and B) and DBP (C and D) identified with *selbal* analysis (A and C) cross-validation in balance selection. The barplots illustrate the stability of the most frequent balanced members during the cross-validation procedure: the blue bars correspond to the numerator members, and orange bars correspond to the denominator members. (B and D) Linear regression between the selected balances and the blood pressure level (SBP and DBP).

The associations between the microbial balance (the normalized log ratio of the geometric abundance means of two groups of taxa) and BP were uncovered by applying a compositionality-aware algorithm*—*selbal (17) (Fig. 2). The genera mostly associated with SBP are *Allobaculum*, *Bacteroides*, and *Alistipes* (Fig. 2A), whose occurrences in the cross-validation procedure are at least 40%. Furthermore, a balance consisting of *Alistipes* and *Erysipelotrichaceae incertae sedis* (as numerators) to *Allobaculum* and *Bacteroides* (as denominators) had a strong correlation with SBP (Fig. 2B, *R*^2^ = 0.762), which indicates that higher levels of *Alistipes* and *Erysipelotrichaceae incertae sedis* were positively associated with SBP, while the relative abundances of *Allobaculum* and *Bacteroides* were negatively associated with SBP. Conversely, a balance represented by the log ratio of the numerators (*Saccharibacteria genera incertae sedis*, *Aminiphilus*, *Methanobrevibacter*, and *Xylanibacter*) and the denominators (*Pyrolobus* and *Hydrogenoanaerobacterium*) had a strong correlation with DBP (Fig. 2D, *R*^2^ = 0.747). The four genera in the numerator group positively affect DBP, while the bacteria in the denominator group negatively affect DBP. *Saccharibacteria genera incertae sedis* was selected in 68% of cross-validation iterations, and *Pyrolobus* was selected in 66% (Fig. 2C). These results indicated that the bacterial interactions are strongly associated with BP, and future studies should assess the co-links of taxa to better understand the possible mechanisms by which bacteria influence BP.

### Identify treatment-specific microbial signatures associated with BP using linear mixed model

**Fig 3:**
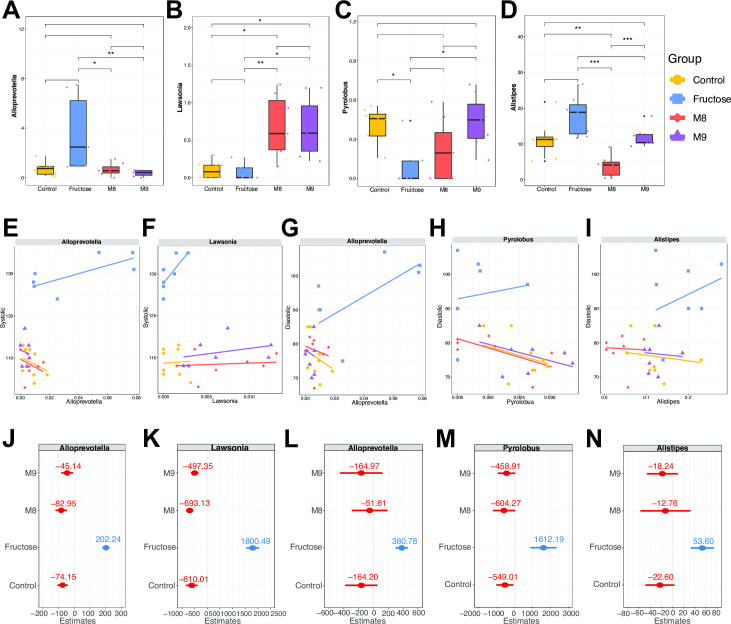
The genera identified as signatures related to blood pressure show different patterns of response to different treatments. (A–D) The relative abundance level of the genera in each group. Wilcoxon test: *P < 0.05, **P < 0.01, ***P < 0.001. (E–I) The abundance of each genus regressed on the SBP (E and F) or DBP (G–I). The colored lines are the fitted regression lines for each group based on the estimated intercepts and slopes. (J–N) Estimated slopes of random factor for each genus model on the SBP (J and K) and DBP (L–N).

We next explored the associations between the taxonomic and functional levels of the gut microbiota and BP with linear mixed models considering treatments as random effects (see Materials and Methods for more details). Four genera (*Alloprevotella*, *Lawsonia*, *Pyrolobus*, and *Alistipes*) whose abundance differed between the probiotic group and the fructose group (Wilcoxon test, FDR-corrected *P* < 0.25) were identified as microbial signatures associated with BP level (Fig. 3A through D). Compared to the fructose group, elevated levels of both *Lawsonia* and depleted *Alloprevotella* were detected in the M8 and M9 groups (Wilcoxon test, FDR-corrected *P* < 0.25). Conversely, *Alistipes* was less abundant only in the M8 group, and *Pyrolobus* was more abundant only in the M9 group (Wilcoxon test, FDR-corrected *P* < 0.25).

The linear mixture models of SBP and DBP regressed on the abundance of each genus regarding the treatments as random effects demonstrated that the treatment-specific effects (random slopes or random regression coefficient) differed among the four groups (Fig. 3E through I; Table S6), indicating that the potential modulation effects of different treatments on BP varied, partially explained by different microbial interactions and dynamics introduced by different treatments. Overall, the relative abundances of *Alloprevotella* and *Lawsonia* were associated with SBP. The genera *Alloprevotella* and *Lawsonia* exhibited positive relationships with SBP in the fructose group (,Fig. 3E, F, J, and K; Table S6) (*Alloprevotella*: coefficient = 97.270, *Lawsonia*: coefficient = 2563.830). In three other groups, *Alloprevotella* showed negative effects on SBP (control: coefficient = −179.119, M8: coefficient = −187.926, and M9: coefficient = −150.108), whereas *Lawsonia* showed positive correlations (Control: coefficient = 153.333, M8: coefficient = 70.213 and M9: coefficient = 265.987). On the other hand, DBP was positively associated with *Alloprevotella*, *Pyrolobus*, and *Alistipes* in the fructose group but negatively associated in three other groups (see Fig. 3F, H, J, L, and N; Table S6).

In terms of microbial function, five Kyoto Encyclopedia of Genes and Genomes (KEGG) pathways, namely, “other glycan degradation (ko00511),” “glycosphingolipid biosynthesis—globo and isoglobo series (ko00603),” “base excision repair (ko03410),” “nucleotide excision repair (NER) (ko03420),” and “D-glutamine and D-glutamate metabolism (ko00471)” were associated with DBP. All five pathways were significantly less abundant in the M8 group than in the fructose group (Wilcoxon test, FDR-corrected *P* < 0.05) (Fig. S4). In each of these pathway models, we observed positive relationships between the pathway abundances and DBP in the fructose group (Fig. S4; Table S6). In the M9 group, other glycan degradation (ko00511) and nucleotide excision repair (ko03420) showed negative relationships (ko00511: coefficient = −4.49, ko03420: coefficient = −9.65) with DBP, while the other three pathways had a positive effect on DBP. In the M8 group, the five pathways all showed a slightly positive effect on DBP. Moreover, in the base excision repair (ko03410) model, the M8 treatment had the greatest negative affect on DBP (random regression coefficient = −55.51). The M9 treatment had the strongest negative effect on DBP in the model of nucleotide excision repair (ko03420) (random regression coefficient = −98.49). The model based on each genus/microbial pathway together suggests that there could be diverse interactions between the gut microbes or microbial pathway and different treatments in altering BP levels. Besides, we observed a higher F4/80 immunoreactivity in the fructose group and a reduction of F4/80 protein-immunoreactive accumulations in the M8 and M9 groups (see Fig. S5). Immunohistochemical staining of F4/80 in colon sections in the fructose group also revealed mucosal damage, notable granulocyte infiltration, and mucosal and muscle hyperplasia compared to other groups (Fig. S5).

### Association analysis between the gut microbes and serum metabolic function

**Fig 4:**
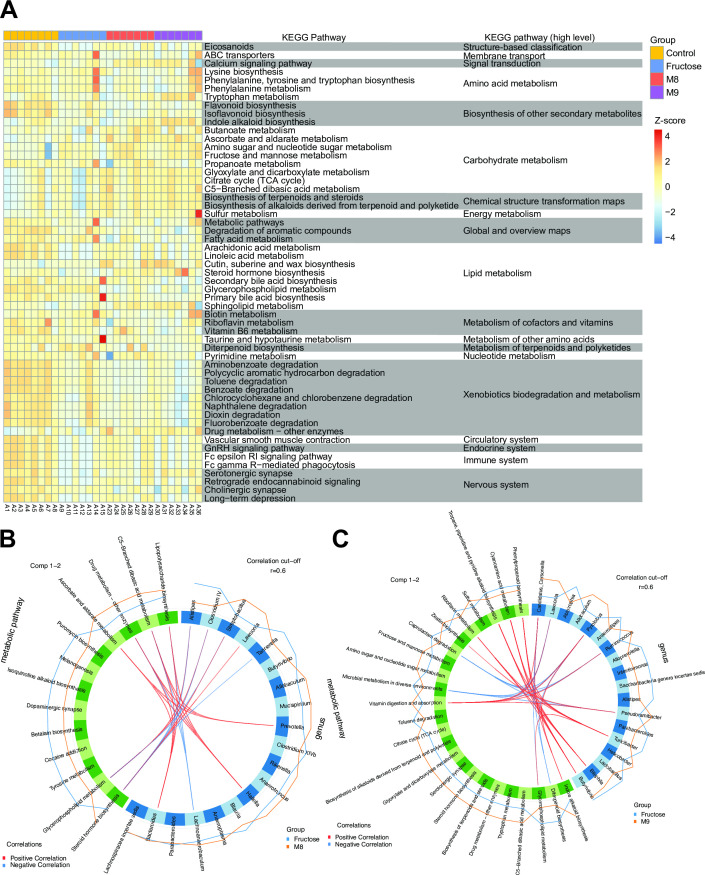
Gut microbiome and serum metabolomics are strongly linked. (A) Heatmap of serum metabolic pathways showing differential activity (measured as pathway activity profiling [PAPi] score) among the four groups. For visualization, the PAPi scores were log-transformed then centered and scaled to a mean of 0 and standard deviation of 4. FDR-adjusted *P* value threshold was set as 0.01. (B and C) Correlations between genus and metabolic pathways identified using Spls-DA. With the correlation cutoff of 0.6 in absolute value, we identified highly correlated genus and metabolic pathways that were discriminative between the probiotics intervention group (M8, B; M9, C) and the fructose group. The orange and blue lines outside the circle represent the average abundance level of each genus (or metabolic pathways) within M8 (or M9) and fructose groups separately. TCA, tricarboxylic acid cycle.

As the metabolites of gut microbiota could influence the host physiology, we analyzed the serum metabolic profiling to explore the differences of host metabolic functions among these groups. A total of 55 KEGG pathways had differential metabolic activities in at least one pairwise comparison among the four groups with FDR of <0.1 (Fig. 4A; Table S7). We found that high-fructose treatment extensively changed the activities of host metabolic pathways, with 20 (34.5%) differential pathways identified. The pathways associated with the circulatory system, nervous system, immune system, and xenobiotics biodegradation and metabolism had a low activity in the fructose group. Notably, the pathways involved in the circulatory system and the nervous system, such as vascular smooth muscle contraction, serotonergic synapse, and cholinergic synapse, are known to be associated with BP regulation (18–20). We further looked into the abundance of important metabolites involved in these pathways. L-Tryptophan, serving as the precursor for 5-hydroxytryptpamine in the serotonergic neurons (21), was significantly increased in the M9 group (Wilcoxon test: fructose vs M9, *P* = 0.002). Another metabolite arachidonic acid that is able to relax blood vessels (22) was found to increase in the M8 group (Wilcoxon test: fructose vs M8, *P* = 0.073).

In addition, decreased “lysine biosynthesis” (44.62% median decline) and “biotin metabolism” (44.62% median decline) were found in the M8 group as compared to the fructose group. When comparing the M9 with the fructose groups, we identified three differential pathways, including elevated levels (38.11% median increase) of “indole alkaloid biosynthesis” and depletion of “diterpenoid biosynthesis” (65.19% median decline) and “secondary bile acid biosynthesis” (69.34% median decline) in the M9 group.

We next adopted sparse partial least squares discriminant analysis (sPLS-DA) analysis to explore the relationships between genus and metabolic pathways under different treatments. Between the M8 and fructose groups, we identified 22 genus-pathway associations (|correlation| > 0.6) consisting of 9 genera and 6 metabolic pathways (Fig. 4B; Table S8). On average, each metabolic pathway was associated with four genera. Steroid hormone biosynthesis was the most connected metabolic pathway, being associated with *Alistipes* (correlation = −0.902), *Clostridium* IV (correlation = −0.869), *Tannerella* (correlation = −0.700), *Streptobacillus* (correlation = −0.760), and *Lawsonia* (correlation = 0.725). *Bacteroides* and *Hallella* were both associated with four metabolic pathways.

On the other hand, 32 genus-metabolic pathway associations (|correlation| > 0.6) consisting 8 genera and 11 metabolic pathways were identified between the M9 and fructose groups (Fig. 4C; Table S9). “Vitamin digestion and absorption” had the highest number of positive associations with different genera, such as *Pseudoramibacter* (correlation = 0.714), *Pyrolobus* (correlation = 0.714), *Lawsonia* (correlation = 0.714), *Ruminococcus* (correlation = 0.669), and *Candidatus Carsonella* (correlation = 0.651). *Pyrolobus* and *Turicibacter* were independently associated with five metabolic pathways. Moreover, the strongest relationship was shown between *Bilophila* and “tropane, piperidine, and pyridine alkaloid biosynthesis” (correlation = 0.884). Notably, indole alkaloid biosynthesis with a significantly higher activity in the M9 group was positively correlated with *Pyrolobus* (correlation = 0.629) and *Candidatus Carsonella* (correlation = 0.604), whose abundance was significantly increased in the M9 group. In summary, M8 tended to modulate (positively or negatively) the host lipid metabolism, while M9 tended to alter the vitamin metabolism and biosynthesis of secondary metabolites, which suggests that the two probiotics may regulate the BP levels via different mechanisms.

## DISCUSSION

In this study, through probiotic intervention experiments on a high fructose-induced hypertensive mice model, we discovered that the probiotics *B. lactis* M8 and *L. rhamnosus* M9 significantly reduced BP levels. Linking the clinical outcome with the altered gut microbiota induced by probiotics, we identified several key microbial taxa, functions, and host metabolic pathways associated with decreased BP. Moreover, we identified diverse associations between specific genera and metabolic functions, which abundances differed significantly among probiotic treatments. In particular, the probiotics reduced the abundance of *Alistipes* and increased the level of *Pyrolobus*, which is associated with lipid and vitamin metabolism, subsequently attenuating the development of hypertension. These results uncovered the role of the gut microbiota in BP regulation, demonstrating the potential therapeutic value of the probiotics in the prevention and auxiliary treatment of hypertension.

We found that the M8 and M9 reduced BP by altering the abundances of several key bacteria. Notably, we revealed that the decreased level of *Alistipes* induced by M8 was correlated with the reduced level of BP, consistent with previous reports that *Alistipes* was positively correlated with BP (23, 24). Moreover, *Alistipes* was associated with the level of bile acid, influencing portal hypertension though farnesoid-X receptor (25, 26); therefore, we speculated that *Alistipes* might affect the host hypertension via bile acid metabolism. Additionally, the strong negative correlation between *Alistipes* and steroid hormone biosynthesis may suggest that *Alistipes* could reduce the BP by regulating steroid hormone biosynthesis, which is associated with hypertension (27). The potential mechanisms underlying the link between *Alistipes* and BP certainly need more evidence based on future well-controlled studies.

Another SCFA‐producing bacterium, *Alloprevotella*, increased its abundance in the high-fructose treatment but decreased in both the treatment with M8 and M9. The increased abundance of *Alloprevotella* in the hypertensive mice was in line with a previous report that the SCFAs have higher levels in the feces of hypertensive individuals (28). In addition, another study showed that *Alloprevotella* associated with the pathogenesis of hypertension by promoting the epithelial inflammatory response (29). Together, these results demonstrated that *Alloprevotella* could modulate BP through regulating the production of SCFAs.

Other SCFA-reducing bacteria, such as *Coprobacillus* and *Butyricimonas*, were both more abundant in the M9 group as compared to the fructose group, which suggests their roles in BP regulation. Previous studies found that *Coprobacillus* can affect human health in various ways, such as by inhibiting fat absorption (30),and reducing immune system reactivation and inflammation (31). These observations, along with our findings, underline the fact that SCFA production is not the only relevant function of these bacteria, and other metabolic capacities of the gut microbiota may also contribute to BP regulation. Therefore, although we did not detect SCFAs in our metabolomics data, the modifications in the abundances of SCFA-producing bacteria still provide valuable insights into the potential mechanisms by which the gut microbiota can influence BP.

We also identified bacteria that were significantly altered in the fructose group as compared to the control group. For example, *Allobaculum* was highly abundant in the control group as compared to the fructose group, and it tended to be more abundant in the M9 group. These results together indicate that *Allobaculum* could prevent high BP, which is consistent with the previous report that BP was highly negatively correlated with the elevated abundance of *Allobaculum* (32, 33). Moreover, considering the role of *Allobaculum* in mucin degradation and its inverse correlation with circulating leptin levels, known to influence BP (34), we speculate that changes in leptin concentration might impact mucin production and, consequently, the composition of the gut microbiota due to the activity of *Allobaculum*.

In the present study, the gut microbes were analyzed at the genus level with RDP classifier. Since RDP classifier is incapable of offering species-level taxonomic profiling, we utilized Kraken2 to assign metagenomic reads at the species level (35). Six species, including *Bacteroides dorei*, *Bacteroides vulgatus*, and *Bifidobacterium animalis*, were more abundant in he M8 group. The other 10 species, such as *Alistipes shahii*, *Alistipes dispar*, and *Alistipes megaguti*, were less abundant in the M8 group, consistent with the significantly reduced levels of *Alistipes* identified by RDP. Among the eight differential species in M9 with fructose, four species were also differentially abundant when we compared M8 with fructose. Specifically, more abundant *Paenibacillus larvae* and less abundant *Lactobacillus reuteri* and *Lactobacillus johnsonii* were found in the M8 and M9 groups as compared to the fructose group. *Bacteroides dorei* was significantly increased in the M8 group but decreased in the M9 group. Notably, a *Lactobacillus reuteri* strain was reported to improve insulin resistance and to facilitate glucose intake in high fructose-fed rats (36). These probiotics-modulated species should be investigated further for their influence on BP.

Functionally, we discovered that the reduced BP in the M8 and M9 groups is associated with the decreased levels of several gut microbial pathways, including BER and NER, which belong to the DNA repair system. Microbiota dysbiosis may lead to an accumulation of unrepaired DNA breaks and BER intermediates that can contribute to hypertension formation (37). The metabolism of D-glutamine and D-glutamate has been found to be correlated with hypertension (38). Another metabolic pathway, glycosphingolipid biosynthesis, has been shown to improve insulin sensitivity, which is a hallmark of metabolic syndrome (39). Together, the extensive changes identified in the microbial pathways triggered by the probiotic treatment were supported by previous findings.

Moreover, the analysis of associations between microbes and metabolome could gain more insights into the specific mechanisms. In terms of the significant correlations of metabolic pathways, steroid hormone biosynthesis (ko00140) is the most connected metabolic pathway in the microbe-metabolite associations between the M8 and fructose groups. Researchers have found that different classes of steroid hormone have a distinct/divergent effect on BP. For instance, an *in vivo* experiment showed that testosterone supplementation and reduction of 17 beta-estradiol activated the renin-angiotensin-aldosterone system to increase BP (40, 41). Additionally, cortisone promoted the reabsorption of water and sodium in the kidneys and increased BP after being transformed into the active hydrocortisone (42). Notably, *Clostridium* spp. have been reported to produce enzymes that modify steroidal hormones (43, 44). Moreover, another study showed that steroidal hormones may participate in the pathogenesis of toxigenic clostridial infections (45). These findings suggest that the bacterial modification to the steroidal hormones plays important role in regulation BP. Combining with our results, we can speculate that the species showing strong correlations with steroid hormone biosynthesis, including *Alistipes*, *Clostridium* IV and *Tannerella*, might contribute to the transformation of steroidal hormones by their metabolic products.

Vitamin digestion and absorption is another important pathway by M9 in the analysis of microbes-metabolites associations. Vitamin is a type of essential nutrients required for growth and immune function (46). Intestinal microbes are able to produce vitamins via enzymatic pathways involved in vitamin synthesis eight different B complex vitamins, such as biotin and cobalamin (47). These microbiota-derived vitamins are primarily absorbed in the colon. Thus we can infer that the microbes showing strong positive correlations with vitamin digestion and absorption, such as *Ruminococcus*, is able to influence colon function via vitamin synthesis pathway, then having an impact on disease state. Additionally, in the IBD patients, metabolic pathway vitamin digestion and absorption and *Ruminococcus* spp. were significantly altered (48). These results supported the importance of this correlation in the disease state, and it is worthwhile to explore the production of different vitamins in *Ruminococcus*.

We also attempted to interpret the connections of the bacteria with metabolic pathways. For instance, *Ruminococcus* spp., regulated by M9, were positively correlated with vitamin digestion and absorption. Notably, *Ruminococcus* has been found to be related to vitamin D biosynthesis (49). Vitamin deficiency is thought to be a risk factor for the development of hypertension (50). It has been shown that the gut microbiota could act as an important supplier or synthesizers of vitamins. *Lactobacillus reuteri* was found to increase the serum vitamin D level (51). Vitamin D plays a role in suppressing renin-angiotensin-aldosterone activity, improving function of vascular wall and alleviating vascular oxidative stress to regulate hypertension (52). Taken together, we identified several genera that potentially regulate BP via vitamin metabolism, and these bacteria warrant further molecular experiments for validation.

Importantly, some metabolic pathways of the circulatory system and nervous system, directly related to hypertension, were altered by *B. lactis* M8 and *L. rhamnosus* M9. Vascular smooth muscle contraction, the key factor to hypertension, was decreased by fructose intake and partially recovered by probiotic treatment, suggesting an improvement of vascular reactivity and tone (18). In the serotonergic synapse pathway, arachidonic acid can relax blood vessels and reduce hypertension (53). Cholinergic synapse regulated by probiotics was involved in the enhanced sympathetic outflow and BP elevation by rostral ventrolateral medulla (54).

In addition, through abundance analysis and association study, we found that *B. lactis* M8 and *L. rhamnosus* M9 regulated hypertension under distinct modulation effects. For example, M8 might prevent the chronic inflammation reflected by F4/80 through increasing the *Bacteroides* abundance, which is negatively associated with lipopolysaccharide biosynthesis, while M9 may regulate tryptophan metabolism to prevent inflammation. Distinctive mechanisms of probiotic action have been observed across taxonomic groups, often with strain specificity. Previous systematic review summarized the disease-specific probiotic efficacy in 70% of probiotic strains among four preventive indications and 65% of probiotic strains among five treatment indications from 228 trials (55). Furthermore, two *Akkermansia muciniphila* strains showed similar anti-inflammatory effects *in vitro* but exhibited the strain-specific properties *in vivo* in mouse models, suggesting that the probiotics might be influenced by the complex gut environment (56). In our case, human milk isolated M8 and M9 exhibited high tolerance to gastric acid and bile salts (57). Moreover, M8 was able to prevent dementia in mice, and M9 had an anti-cancer effect via regulating gut microbiota or metabolome (58–60). In clinical studies, M8 protected against acute respiratory tract infections in children and regulated gut dysbiosis in Parkinson patients, and M9 alleviated stress in humans (61–63). More studies are needed to investigate their modulation effect, thereby providing guidance for clinical application.

This study has several limitations. First, the small sample size may lead to an insufficient statistical power to detect the small difference caused by the probiotic treatment. Moreover, to further illustrate the efficacy of probiotics, more factors should be considered, such as treatment duration and the form and dosage of probiotic treatment. Additionally, exploring the interaction and synergistic effect of the two probiotics is necessary to develop an appropriate combinatorial therapy, which may enhance the efficacies against hypertension. Compared to drug therapy, dietary probiotic supplements are useful alternatives to alleviate hypertension via a less radical approach.

### Conclusions

*B. lactis* M8 and *L. rhamnosus* M9 could prevent the hypertension, and such process was related to the modulation of the gut microbiota and host metabolic activity. The probiotics-reduced BP was associated with the increased *Lawsonia* and *Pyrolobus* and reduced *Alistipes* and *Alloprevotella*. Further association analysis between microbial taxa and host metabolic activities revealed that *Alistipes* could potentially modulate BP by affecting steroid hormone levels. Moreover, M8 modulated the bacteria involved in lipid metabolism, while M9 altered the bacteria-associated vitamin metabolism and biosynthesis of secondary metabolites. Both strains regulated metabolic pathways including vascular smooth muscle contraction, serotonergic synapse, and cholinergic synapse. These findings would deepen our understanding of the modulation effects of probiotics on the gut microbial composition and hypertension adjustment, paving the way for future human interventional studies for alternative therapeutic options.

## MATERIALS AND METHODS

### Animals and housing

Mice were kept under a standard controlled condition (12-hour light/dark cycle; temperature, 22°C ± 2°C; humidity, 55% ± 5%) with free access to food and water during the 1-week acclimatization period after delivery to the animal house of our laboratory. After the acclimatization week, these 8-week-old male mice were randomly assigned into four groups (*n* = 8 per group, housed in groups of four mice per cage). All four groups of mice were fed normal diet. The control mice drank normal water; the other three groups of mice drank 20% (wt/vol) high-fructose water. Meanwhile, mice in the probiotic intervention groups were supplemented with probiotic (*Bifidobacterium lactis* M8 or *Lactobacillus rhamnosus* M9) with a daily oral gavage of 4 × 10^9^ CFU in 0.1 mL for 16 weeks. All animals in the control and model groups (LI) received the same volume of saline by oral gavage in place of probiotics. At the end of the experiment, mice were sacrificed by decapitation under anesthesia and allowed to die within a short period to minimize pain. The mice fasted for 12 hours the day before they were sacrificed. Stool and serum samples were collected from all mice and stored at −80°C. The systolic/diastolic blood pressures of animals were measured at 9:00–10:00 a.m. by indirect tail-cuff IITC blood pressure system (IITC/Life Science Instruments, USA).

### Serum metabolic profiling

#### MS analyses of polar metabolites in serum with positive ion mode

Liquid chromatograph mass spectrometer (LC-MS) samples were prepared from serum (100 µL) mixed with four volumes of 1:1 (vol/vol) methanol/acetonitrile. The samples were centrifuged (15 minutes, 17,000 *g*, 4°C), and 5 µL of supernatants was injected directly onto a 2.1 × 100 mm ACQUITY UPLC HSS T3 Column (Waters). The column was eluted isocratically at a flow rate of 400 µL/min with a gradient from 95% to 40% mobile phase A (0.1% formic acid in water) and a gradient from 5% to 60% mobile phase B (0.1% formic acid in acetonitrile) over 10 minutes, then followed by a 5% mobile phase A and 95% mobile phase B for 2 minutes and another 2 minutes at a 95% A and 5% B. MS analyses were carried out under auto MS/MS mode (LC/MS Data Acquisition, version B.08.00; Waters) using electrospray ionization in the positive ion mode using full scan analysis over *m*/*z* 50–1,100 at 70,000 resolution and 3-Hz data acquisition rate. Additional MS settings were ion spray voltage, 4.0 kV; capillary temperature, 350°C; probe heater temperature, 320°C; sheath gas flow, 12 L/min; and auxiliary gas flow, 8 L/s.

#### MS analyses of polar metabolites in serum with negative ion mode

The preparation of LC–MS samples, the injected column, the eluted program, and the MS settings were the same as mentioned above. The difference is that these were carried out in the negative ion mode of electrospray ionization with a 3.5-kV ion spray voltage; the composition of mobile phase A is 2 mM ammonium acetate in water, and mobile phase B is acetonitrile. The pathway activity profiling algorithm was used to compare the activities of metabolic pathways from metabolic profiles under different conditions (64).

### Immunohistochemical staining

Paraffin-embedded tissues (colon and kidney) were sectioned (5 µm) and the slides were placed in 0.01 mM citrate buffer (pH 6.0) for antigen retrieval (at 120°C for 10 min). Then, blocking with goat serum (Beyotime, China) was performed for 60 minutes. The slides were incubated with primary antibodies including rabbit polyclonal to F4/80 (Abcam) overnight at 4°C. Next, goat anti-rabbit IgG was added and incubated with SABC-HRP Kit (Beyotime). Finally, slides were visualized with DAB compound (Beyotime) and viewed on a Leica Aperio CS2 system (Germany).

### Metagenomic data quality control, assembly, gene prediction, and functional annotation

The high-quality reads were obtained by removing the adapter regions, low-quality reads and PCR-duplicated reads as previously described (65, 66). This step was implemented by using the script at https://github.com/TingtZHENG/VirMiner. These reads were subsequently mapped to the mice reference genome to filter out the host contamination using Burrows-Wheeler aligner, and the mapped reads identified with identity 99% were discarded (67). IDBA_UD was used to do the *de novo* assembly based on paired-end reads with default parameters (68). Gene annotation on all the assembled contigs were performed using hidden Markov models from MetaGeneMark (69). The gene abundances were measured using reads per kilobase per million mapped reads. The predicted open reading frames (ORFs) were annotated to KEGG orthology (KO) using BLASTP with an *e*-value cutoff of 1e-5 (70). Then KEGG pathways were identified based on these KO entries.

### Taxonomic assignment and diversity analysis

The reads were assigned to different taxonomies, and the taxonomic relative abundances were calculated using RDP native Bayesian classifier (71). Alpha diversity (measured as Shannon index), species richness (measured as Chao1 richness), and beta diversity (measured as weighted UniFrac distance) were calculated at genus level. These calculations were implemented by using R package vegan (72).

### Explore the associations between microbial taxa and blood pressure based on compositional balance analysis

The selbal algorithm proposed by Rivera-Pinto et al. was used to identify microbial taxa associated with blood pressure, with default parameters (17). The genera present in over 50% samples were used. The SBP and DBP were considered as the outcomes in the selbal procedure, respectively.

### Identify microbial signatures associated with blood pressure using linear mixed model

The genera/microbial pathways used for linear mixed model were first preselected using the rfe function from caret package (73) (The R Foundation; https://cran.r-project.org/web/packages/caret/). Afterward, each genus/microbial pathway was used to fit a linear mixed model, which regressed each genus abundance on the SBP or DBP separately and allowed each genus to have different patterns of response to the treatment groups. The probiotic effect was defined as the effect that reversed the changes in each genus/microbial pathway induced by fructose, which was quantified by the difference in estimated random regression coefficients (random slope) between the M8 (or M9) and fructose groups. The significance of the probiotic effect was calculated with the permutation tests by shuffling the M8 (or M9) and fructose labels. Ultimately, a genus/microbial pathway is determined as signature if it meets the following criteria: (i) significance level of probiotic effect less than 0.05 and (ii) being listed as differentially abundant genus/microbial pathway in the comparison of M8 vs fructose or M9 vs fructose.

The linear mixed models were implemented by lmer function from the lme4 R package (74), with the formula as lmer [blood pressure (systolic or diastolic) ~ genus + (1 + genus|group), data = data set]. The fixed effect and random effect from these models were extracted with the lme4 function fixef and ranef. The estimated slopes of random factor were plotted with the “plot_model” function from the sjPlot R package.

### Associate genus-level abundance matrix with metabolic pathway profiling using sPLS-DA

First, sPLS-DA implemented in the MixOmics R package was employed to identify associations of genera present in at least 50% in all the samples and gut metabolic pathways (75). The grouping information was used as the outcome. The number of selected variables to be retained on each component was chosen over the grid of values, ranging from 4 to 15 with an interval of 1 for the gut genus, ranging from 4 to 25 with an interval of 1 for the metabolic pathways. Visualization was implemented with circosPlot.

### Comparative statistical analysis

Statistical test on pairwise comparisons of alpha diversity, species richness measurements, the relative abundance of genera, the abundance of microbial functions, and the activity score of metabolic pathways were performed using Wilcoxon test by R. Multiple testing was corrected using the Benjamini and Hochberg method. Adonis test was used to detect the microbial community composition difference.

## Data Availability

Our study contains metagenome data. Metagenome data were uploaded to the public National Genomics Data Center (https://ngdc.cncb.ac.cn/), Genome Sequence Archive (GSA) data sets; GSA project ID is CRA009575.

## SUPPLEMENTAL MATERIAL

Figure S1 msystems.00331-23-s0001.pdf Blood pressure level change across time points.

Figure S2 msystems.00331-23-s0002.eps Taxonomic composition at the phylum level in the gut microbiota.

Figure S3 msystems.00331-23-s0003.pdf Significantly altered species of the gut microbiota community.

Figure S4 msystems.00331-23-s0004.pdf The microbial pathways identified as signatures related to blood pressure show different patterns of response to different treatments.

Figure S5 msystems.00331-23-s0005.eps Representative images of F4/80 immunohistochemically stained colon sections.

Legends msystems.00331-23-s0006.doc Supplemental legends.

Additional supplemental tables msystems.00331-23-s0007.xlsx Tables S7 to S9.

Supplemental tables msystems.00331-23-s0008.xlsx Tables S1 to S4.

Table S5 msystems.00331-23-s0009.xlsx Relative abundance profiles of gut microbial pathways.

Table S6 msystems.00331-23-s0010.xlsx The estimated coefficients (slopes) in each linear mixed model based on the genera and microbial pathways.

ASM does not own the copyrights to Supplemental Material that may be linked to, or accessed through, an article. The authors have granted ASM a non-exclusive, world-wide license to publish the Supplemental Material files. Please contact the corresponding author directly for reuse.
