# Supplementary figures and images for: Probiotics Bifidobacterium lactis M8 and Lactobacillus rhamnosus M9 prevent high blood pressure via modulating the gut microbiota composition and host metabolic products
Source: mSystems. 2023 Oct 19;8(6):e00331-23. doi: 10.1128/msystems.00331-23 (PMC10734487; doi:10.1128/msystems.00331-23)

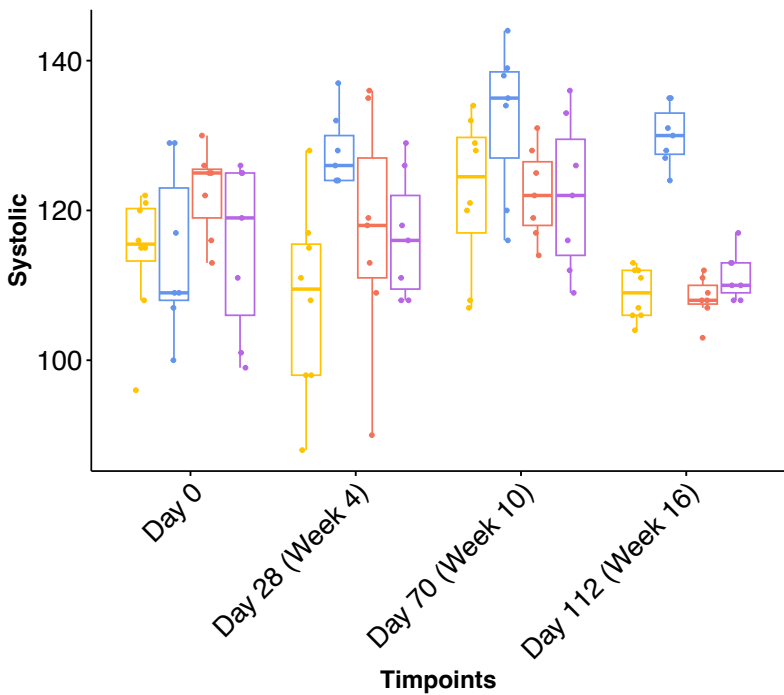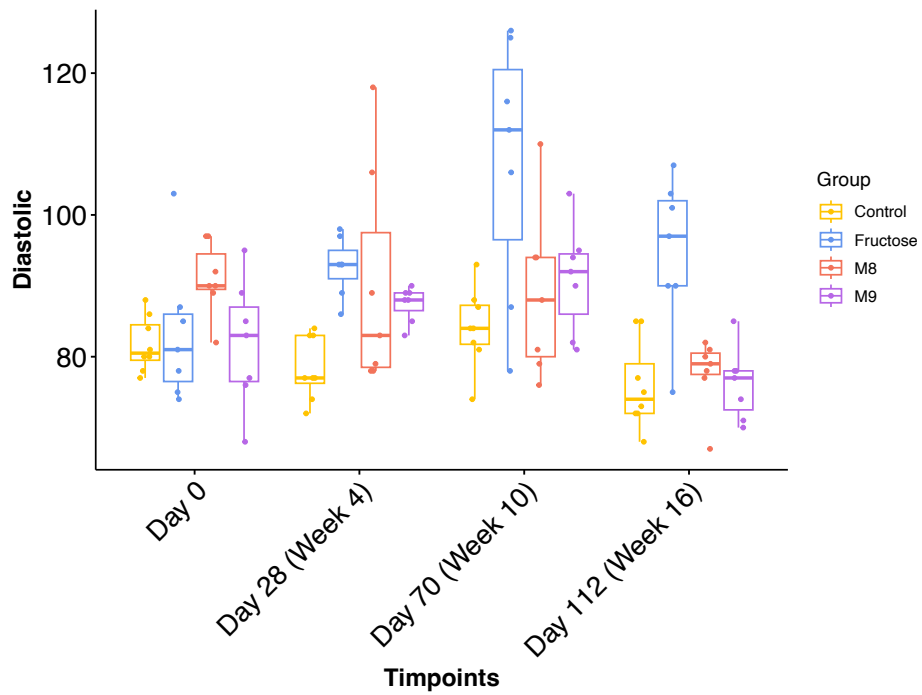

Supplement: Figure S1 — Blood pressure level change across time points. [file msystems.00331-23-s0001.pdf]

**A**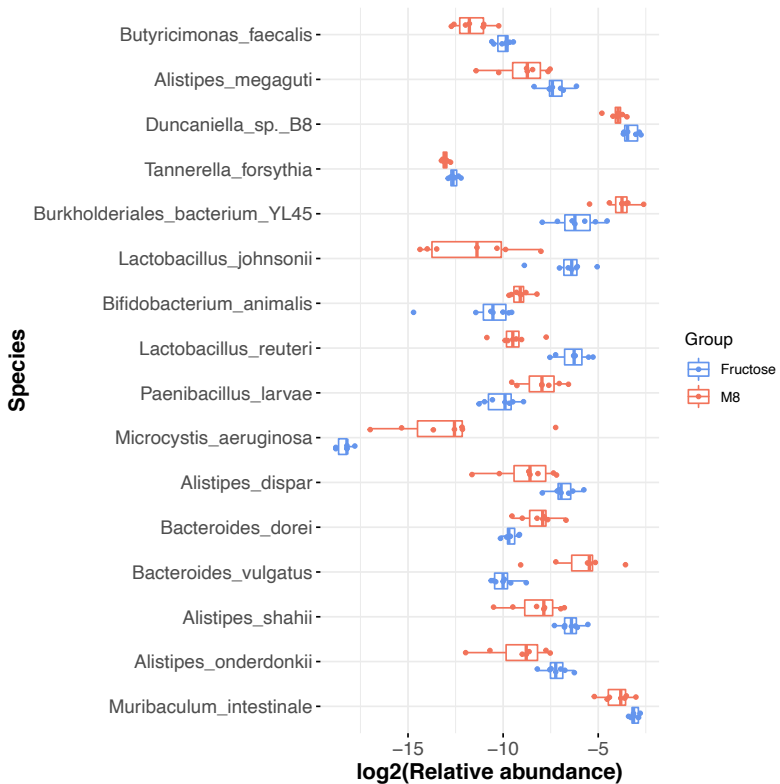**B**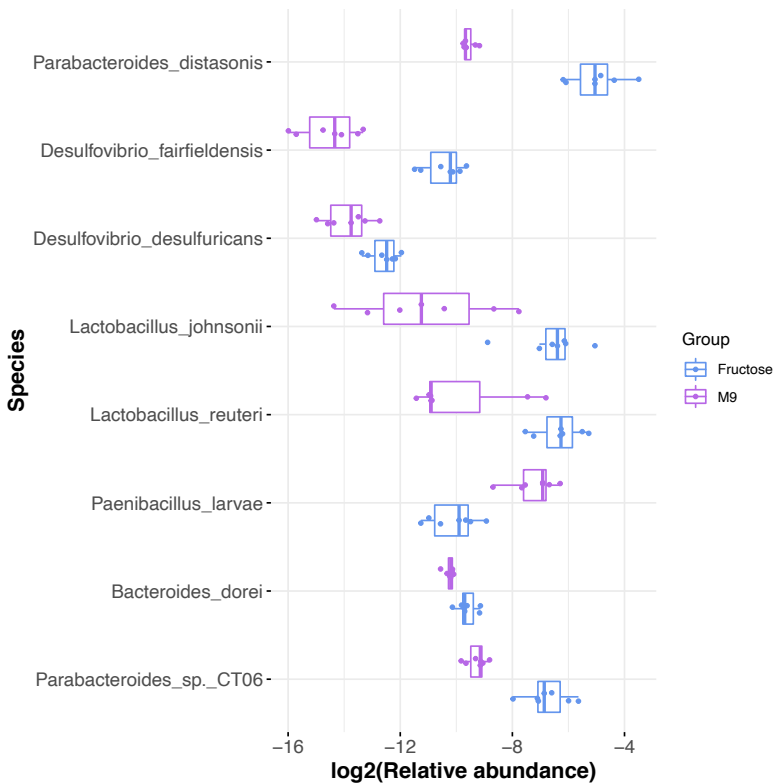

Supplement: Figure S3 — Significantly altered species of the gut microbiota community. [file msystems.00331-23-s0003.pdf]

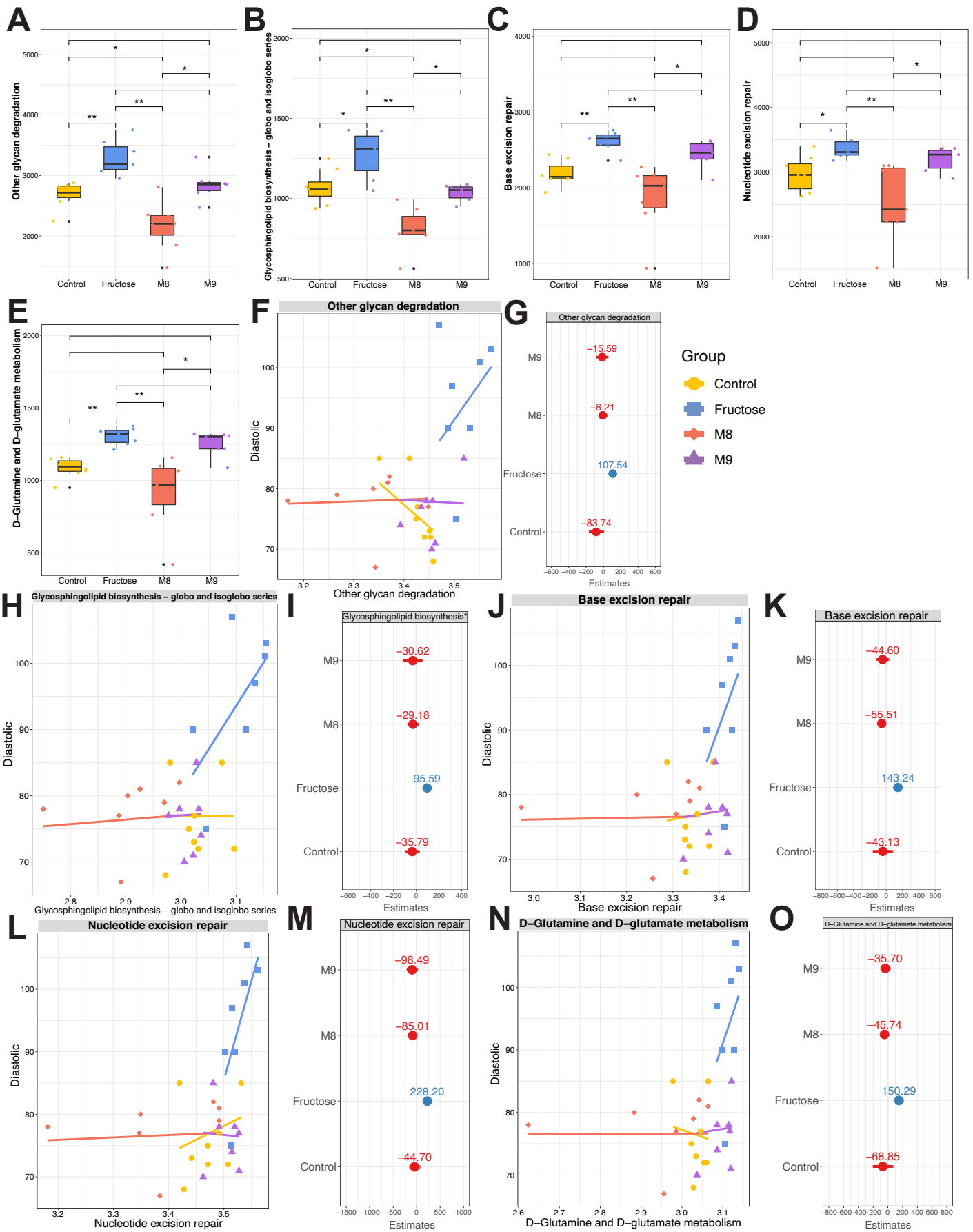

Supplement: Figure S4 — The microbial pathways identified as signatures related to blood pressure show different patterns of response to different treatments. [file msystems.00331-23-s0004.pdf]
