# Supplementary material for: Probiotics Bifidobacterium lactis M8 and Lactobacillus rhamnosus M9 prevent high blood pressure via modulating the gut microbiota composition and host metabolic products
Source: mSystems. 2023 Oct 19;8(6):e00331-23. doi: 10.1128/msystems.00331-23 (PMC10734487; doi:10.1128/msystems.00331-23)
Supplement: Legends — Supplemental legends. [file msystems.00331-23-s0006.doc]

**Supplemental information**

**Figure S1. Blood pressure level change across timepoints**

The systolic and diastolic blood pressure were measured at multiple timepoints, including day 0, week 4, week 10 and week 16.

**Figure S2. Taxonomic composition at the phylum level in the gut microbiota.**

The mean relative abundances are displayed for each phylum in each group.

**Figure S3. Significantly altered species (FDR-corrected *p* < 0.1, mean abundance > 0.01%)** **of the gut microbiota community in the comparison of M8 *vs.* Fructose (A) and M9 *vs.* Fructose (B).**

**Figure S4. The microbial pathways identified as signatures related to blood pressure show different patterns of response to different treatments.**

A-D: the relative abundance level of the microbial pathways in each group. Wilcoxon test: * p < 0.05; ** p<0.01; *** P<0.001. E, G, I, K, M: The abundance of each pathway regressed on the SBP (E, G) or DBP (I, K, M). The colored lines are the fitted regression lines for each group based on the estimated intercepts and slopes. F, H, J, L, N: Estimated slopes of random factor for each genus model.

**Figure S5. Representative images of F4/80 immunohistochemically stained colon sections in four groups (A, E, Control group; B, F, Fructose group; C, G, M8 group; D, H, M9 group; scale bar=600μm or 200μm).**

**Table S1. The blood pressure measured for each mouse used in this study.**

**Table S2. The taxonomic abundance profiles of the gut microbiomes at phylum level.**

**Table S3. The taxonomic abundance profiles of the gut microbiomes at genus level.**

**Table S4. The taxonomic abundance profiles of the gut microbiomes at species level identified with Kraken2.**

**Table S5. The relative abundance profiles of gut microbial pathways.**

**Table S6. The estimated coefficients (slopes) in each linear mixed model based on the genera and microbial pathways respectively.**

**Table S7. The pathway activity (measured as PAPi score) profiles of the KEGG pathways identified in serum metabolomics.**

**Table S8. Feature correlations among gut species and serum metabolic pathways between the M8 and Fructose groups identified by Spls-DA.**

**Table S9. Feature correlations among gut species and serum metabolic pathways between the M9 and Fructose groups identified by Spls-DA.**
